# Expression of microRNAs in bovine and human pre-implantation embryo culture media

**DOI:** 10.3389/fgene.2014.00091

**Published:** 2014-04-24

**Authors:** Jenna Kropp, Sana M. Salih, Hasan Khatib

**Affiliations:** ^1^Department of Animal Sciences, University of Wisconsin-MadisonMadison, WI, USA; ^2^Department of Obstetrics and Gynecology, University of Wisconsin-MadisonMadison, WI, USA

**Keywords:** microRNA, embryonic development, culture media, blastocyst, degenerate embryo

## Abstract

MicroRNAs (miRNA) are short non-coding RNAs which act to regulate expression of genes driving numerous cellular processes. These RNAs are secreted within exosomes from cells into the extracellular environment where they may act as signaling molecules. In addition, they are relatively stable and are specifically expressed in association to certain cancers making them strong candidates as biological markers. Moreover, miRNAs have been detected in body fluids including urine, milk, saliva, semen, and blood plasma. However, it is unknown whether they are secreted by embryonic cells into the culture media. Given that miRNAs are expressed throughout embryonic cellular divisions and embryonic genome activation, we hypothesized that they are secreted from the embryo into the extracellular environment and may play a role in the developmental competence of bovine embryos. To test this hypothesis, bovine embryos were cultured individually from day 5 to day 8 of development in an *in vitro* fertilization system and gene expression of 5 miRNAs was analyzed in both embryos and culture media. Differential miRNA gene expression was observed between embryos that developed to the blastocyst stage and those that failed to develop from the morula to blastocyst stage, deemed degenerate embryos. MiR-25, miR-302c, miR-196a2, and miR-181a expression was found to be higher in degenerate embryos compared to blastocyst embryos. Interestingly, these miRNAs were also found to be expressed in the culture media of both bovine and human pre-implantation embryos. Overall, our results show for the first time that miRNAs are secreted from pre-implantation embryos into culture media and that miRNA expression may correlate with developmental competence of the embryo. Expression of miRNAs in *in vitro* culture media could allow for the development of biological markers for selection of better quality embryos and for subsequent successful pregnancy.

## Introduction

MicroRNAs (miRNAs) are a class of RNA molecules that have recently received a considerable amount of attention due to their remarkable impact on gene regulation. MiRNA molecules are non-coding RNAs that are 18–22 nt in length which act to bind target mRNA to regulate gene expression (Lau et al., 2001; Lim et al., 2003). More specifically, the 5′ end of miRNAs binds within a recognition sequence known as the seed region made up of 2–8 nucleotides found in the 3′UTR of a target mRNA. Binding of the mature miRNA to the mRNA occurs within ribonucleotide protein complexes or RNA-induced silencing complexes (RISC) in the cytoplasm where the miRNA then acts to destabilize or repress translation of the mRNA. MiRNAs are considered negative regulators of gene expression and have been found to act on vast number of genes due to the imprecise binding to a target mRNA (Ambros, 2004; Farh et al., 2005; Filipowicz et al., 2008). Due to the promiscuous nature of miRNA binding, one miRNA can act to regulate over 100 target genes (Lim et al., 2005). Due to the magnitude of impact on expression, understanding miRNAs regulation and action is of growing interest.

Many miRNAs have been found to be associated to the regulation of genes in a tissue- and stage- specific manner (Farh et al., 2005; Wang et al., 2013). Several biologic processes have been identified in which miRNAs have a role such as cell proliferation, cell differentiation, apoptosis, metabolism, signal transduction, development, and cancer (Ambros, 2004; Filipowicz et al., 2008; Hossain et al., 2012; Wang et al., 2013). Lim et al. (2005) illustrated that preferentially expressed miRNAs in cells or tissues may be responsible for regulating a specific gene expression profile to define a given cell type. It has been found that miRNAs are located within the genome at cancer-associated regions where genetic alterations may cause dysregulation within the cell to create a disease state (Wang et al., 2013). Interestingly, miRNAs themselves are regulated epigenetically with about 50% of miRNAs associated with methylation associated CpG sites (Wang et al., 2013). Aberrant expression of miRNAs themselves could thus lead to an increase or a decrease of a target gene leading further to differential expression. Considering the vast number of targets and roles of miRNAs in various cellular processes, further characterization of specific miRNA roles and regulation is warranted.

More recently it has been shown that miRNAs are present not only within cells but also in body fluids such as saliva, urine, and blood plasma (Wang et al., 2010; Rayner and Hennessy, 2013; Xu et al., 2013). Studies have shown that miRNAs are selectively secreted from cells; however, they may be secreted in various forms such as exosomes or apoptotic bodies and often are associated with a lipid or protein carrier (Rayner and Hennessy, 2013; Xu et al., 2013). Chen et al. (2008) tested blood serum samples and found miRNAs to be highly stable, withstanding extreme environment conditions such as freezing and thawing thus offering greater appeal as biomarkers. Moreover, a panel of miRNA markers was identified for various cancers such as prostate, lung, and colorectal cancer (Chen et al., 2008; Mitchell et al., 2008). Indeed, Mitchell et al. (2008) found that an individual's serum sample analyzed for miRNA expression could detect cancer with 60% sensitivity and 100% specificity based on miRNA biomarkers thus further proving the effectiveness miRNAs as biomarkers.

Similar to cancer, embryonic growth is a proliferative process where many genes are tightly regulated for coordinated expression throughout developmental processes. Several studies have illustrated dynamic changes of miRNA expression in both gametes and early embryonic development of mammalian species (Tang et al., 2007; Tesfaye et al., 2009; Hossain et al., 2012; Mondou et al., 2012; Abd El Naby et al., 2013). In mammalian *in vitro* production systems, development is often assessed by morphological criteria as set by the International Embryo Transfer Society or IETS (Van Soom et al., 2003). McCallie et al. (2010) identified miRNA expression differences between embryos of similar morphology that were derived from different fertile donor oocytes and those derived from patients with infertility, such as male factor or polycystic ovary syndrome. More stringent biomarkers to predict embryo quality would allow for better selection of embryos transferred into recipients for a successful pregnancy. Thus, the objectives of this study are to determine if there is an association between the quality of the embryo and miRNA expression and to assess the presence of miRNAs within the *in vitro* culture media from human and bovine embryos.

A panel of candidate miRNAs was chosen based on known roles in embryo development and analyzed for gene expression within bovine embryos and culture media. MiR-25 was chosen as it is dynamically expressed within bovine embryos where expression increases from the 16-cell to the blastocyst stage (Tesfaye et al., 2009). Recently, miR-25 has been shown to mediate several processes such as oxidative stress in primary cardiomyctes (Varga et al., 2013), apoptosis in human ovarian cancer (Zhang et al., 2012) and cell reprogramming (Lu et al., 2012). MiR-181 has been associated with roles in genes relating to cancer (Neel and Lebrun, 2013), immune function through NK cell development (Cichocki et al., 2011) and embryonic development (Lingenfelter et al., 2011). Specifically, miR181-a is present in both bovine oocytes and embryos with increased expression in early stages of development then drops to low levels in the blastocyst and is thought to regulate nucleoplasmin2 a protein important in nuclear organization (Lingenfelter et al., 2011). Evidence across species suggests that the miR-196 plays a key role in regulating HOX genes which encode transcription factors vital to embryonic development (Chen et al., 2011). In bovine, miR-196a is believed to regulate newborn ovary homeobox gene (NOBOX), a transcription factor, implicated in the early bovine embryo development (Tripurani et al., 2011). Additionally, the polymorphism miR-196aCC is associated with spontaneously- aborted fetuses in humans (Jeon et al., 2012). Human blastocyst miRNA characterization found miR-302c to be highly expressed in blastocysts by Rosenbluth et al. (2013). Functionally, the miR-302 cluster has been associated with cellular reprogramming where iPS cells overexpressing miR-302 exhibited suppressed MBD2 expression which in turn increased expression of NANOG (Lee et al., 2013). Another candidate of interest is miR-370 which has a role in regulating the expression of the DNA methyltransferase 3a (Dnmt3a) gene (Liu et al., 2013). Assessment of *in vitro* culture media for the presence of miRNAs may allow for the development of non-invasive biomarkers associated with embryo quality.

## Materials and methods

### Bovine *in vitro* maturation and fertilization of embryos

*In vitro* production of embryos was carried out as described by Driver et al. (2013). In brief, oocytes were aspirated from 2 to 8 mm follicles from ovaries derived from a local slaughter house. Oocytes were matured in M-199 media supplemented with gonadotropins (FSH, LH, and estradiol), gentamicin, sodium pyruvate and 10% fetal bovine serum. After incubation of oocytes for 20 h, they were washed with Tyrode's albumin lactate pyruvate (TALP)-Hepes buffer and 10 cumulus oocyte complexes were transferred to 44 μL drops of fertilization media. Fertilization media consisted of IVF-TL (Millipore, Phillipsburg, NJ) supplemented with sodium pyruvate, gentamicin, and fatty-acid-free bovine serum albumin (BSA). The oocytes were fertilized with frozen-thawed semen with sperm concentration determined by percol sperm separation technique as described by Parrish et al. (1995). The oocytes were fertilized with a final concentration of 1 million sperm/ml. The day of fertilization was deemed as day 0 of culture. Once fertilized, the presumptive zygotes were incubated for 24 h until the day of culture day 1.

### Bovine *in vitro* culture of embryos

**Figure 1:**
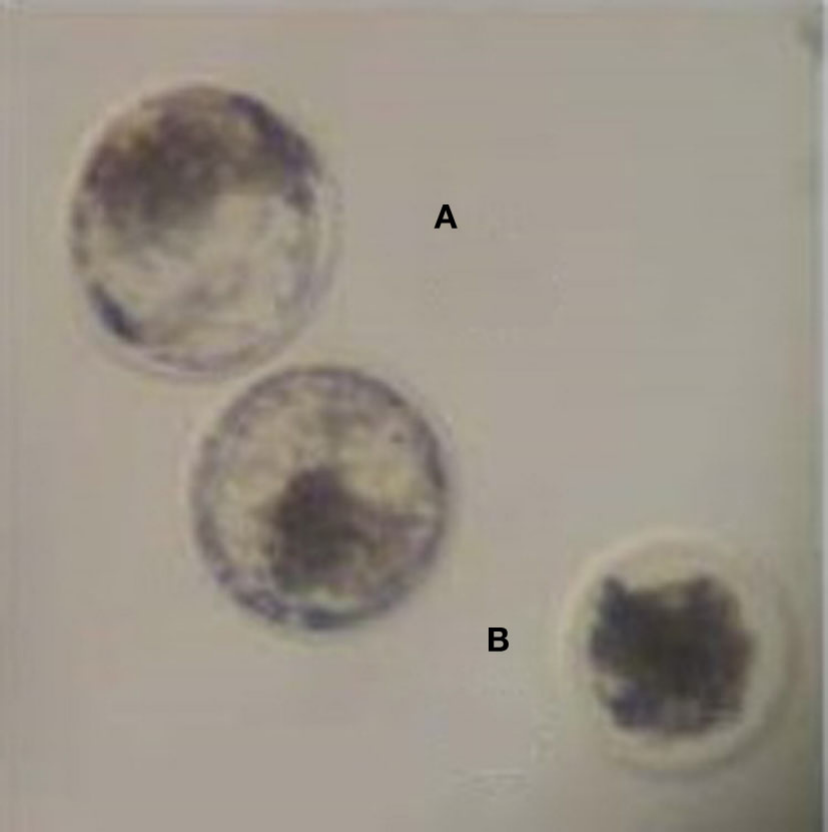
**Day 8 morphological assessment of embryos. (A)** Blastocyst embryos. **(B)** Degenerate embryo.

Zygotes were stripped of their cumulus cells, washed once in TALP-Hepes buffer and cultured in groups of 25 in 50 μL drops of SOF media (Millipore) supplemented with sodium pyruvate, gentamicin, and FAF-BSA. On day 5 of culture, embryos were morphologically assessed and those that had compacted cells characteristic of a morula were transferred to a new drop of SOF media and individually cultured. In Experiment A, day 5 morula were grown in the presence of SOF containing BSA, whereas in Experiment B morula were transferred to SOF lacking BSA supplementation. On day 8, embryos were assessed for development and morphology. Embryos that developed to blastocysts were pooled together, while embryos that failed to develop to blastocysts by day 8 were deemed degenerate, and were pooled separately (Figure 1).

Embryo and media pools were generated within two experiments, across two replicates the SOF media was supplemented with BSA (experiment A), while one replicate was generated for SOF media not supplemented with BSA (experiment B) between day 5 to day 8 of development (Table 1). Two to three pools consisting of growing blastocysts, degenerate embryos, and culture media were collected for each experiment. In experiment A, three pools of blastocysts (pool 1 = 25, pool 2 = 25, and pool 3 = 33), three pools of degenerate embryos (pool 1 = 25, pool 2 = 10, and pool 3 = 19) and one pool of media from embryos in pool 2 were collected. In experiment B, embryos were transferred to SOF culture lacking BSA supplementation and grown individually from day 5 until day 8. Two pools of blastocysts (pool 1 = 22, pool 2 = 21), two pools of degenerate embryos (pool 1 = 17, pool 2 = 19), and two pools of embryos culture media were collected. Across both experiments, control SOF media consisting of media drops that were plated in the same culture petri dish but did not contain embryos was collected.

**Table 1:** **Bovine embryo development and pool design**.

**Experiment**	**Bull ID**	**Total oocytes**	**Fertilization rate (%)**	**Blastocyst rate (%)**		**Embryo (*n*) per pool 1**	**Embryo (*n*) per pool 2**	**Media drops (*n*) per pool 1**	**Media drops (*n*) per pool 2**
A (+BSA)	9939	624	64.31	20.37	B:	25	–	25	30
					D:	25	–	25	25
A (+BSA)	9937	418	61.86	29.44	B:	25	33	33	–
					D:	10	19	19	–
B (−BSA)	9937	437	78.302	24.18	B:	22	17	22	17
					D:	21	19	21	19

### *In vitro* culture media derived from human embryos

Collection of embryo culture media from human *in vitro* fertilization cycles was approved by the Ethics Review Board of the University of Wisconsin-Madison. Embryo culture fluid was collected from reproductive-age women undergoing oocyte retrieval for *in vitro* fertilization. Signed consent for use of fluid from cultured embryos was obtained from each patient. Pronuclear to blastocyst stage embryos were cultured in sequential media system using G-1™ or G-2™ culture media (Vitrolife, San Diego, CA) (Gardner et al., 1998). Prior to embryo placement in new culture drops, embryos were washed in the new medium to remove toxins and from the previous medium. Fertilized embryos were monitored for proper development and graded according to established guidelines (Gardner et al., 2000; Cutting et al., 2008). Embryo culture fluid was collected from singly-cultured day 5 or 6 embryos in addition to control media. Embryo culture fluid from each sample type was placed into RNALater (Ambion, TX) and kept at −80°C until use.

### miRNA extraction

MiRNA was extracted from bovine blastocysts and degenerate embryos using the RNaqueous-Micro kit (Ambion). Isolation of miRNAs of culture media samples was carried out using a cell free specific kit, the miRNeasy Serum/Plasma kit (Qiagen, Germantown, MD). Input volume of each culture media sample for extraction was 200 ul. The Caenorhabditis elegans (C. elegans) miR-39 mimic was added to each extraction as an internal spike-in control using miRNeasy Serum/Plasma Spike-In Control (Qiagen). The concentration of extracted RNA was quantified using NanoDrop ND-1000 spectrophotometer (NanoDrop Technologies, Wilmington, Delaware).

### cDNA synthesis of mirna

Extracted miRNA from both embryos and culture media samples was reverse transcribed into cDNA by the Miscript II RT kit (Qiagen). For subsequent quantification of both mRNAs and miRNAs, the Hi-Flex Buffer was used to generate cDNA from each embryo sample. The maximum input of 10 ul of template was used for each embryo pool due to low starting concentration. In contrast, for each culture media sample, the Hi-Spec Buffer was used for subsequent quantification of mature miRNAs. Reverse transcriptase-PCR reactions were incubated at 37°C for 60 min then at 95°C for 5 min per kit recommendation.

### Quantitative real-time PCR (qRT-PCR) of miRNA

Gene expression analysis was carried out using the miScript SYBR Green Kit (Qiagen). Each embryo cDNA reaction was diluted 1:5 and combined with 2X QuantiTect SYBR Green PCR master mix. For each candidate miRNA, a universal primer specific for miRNAs and a miScript primer assay specific to the miRNA of interest were added. Specific miRNA primers were designed from human mature miRNA sequences and obtained from Qiagen. The glyceraldehyde phosphate dehydrogenase (*GAPDH*) gene was used as an internal control because of its stability across embryo samples (Driver et al., 2013). The primer sequences for both embryo and culture media samples are shown in Table 2. The qRT-PCR reactions were carried out in a Bio-Rad iCycler real-time PCR machine with the following cycling conditions: 95°C for 15 min followed by 40 cycles of 94°C for 15 s, 55°C for 30 s, and 70°C for 30 s.

**Table 2:** **qRT-PCR primer sequences**.

**Gene**	**Assay name (Qiagen)**	**Primer assay for mature miRNA sequence (5′–3′)**
*miR-25*	Bt_miR-25_1	CAUUGCACUUGUCUCGGUCUGA
*miR-302c*	Hs_miR-302c_1	UAAGUGCUUCCAUGUUUCAGUGG
*miR-196A1*	Hs_miR-196a_2	UAGGUAGUUUCAUGUUGUUGGG
*miR-181A1*	Hs_miR181a_2	AACAUUCAACGCUGUCGGUGAGU
*miR-370*	Hs_miR-370_1	GCCUGCUGGGGUGGAACCUGGU
miR-39^*^	Ce_miR-39_1	UCACCGGGUGUAAAUCAGCUUG
*NOBOX* (Tripurani et al., 2011)	Forward:	AGACCCAGTTCTTCCAACACC
	Reverse:	CACAGGACAAGGCAAAGAGAG
GAPDH^**^	Forward:	TGCCCAGAATATCATCCC
	Reverse:	AGGTCAGATCCACAACAG

* *Housekeeping gene/internal control for culture media*.

** *Housekeeping gene for embryos*.

### Statistical analysis of bovine embryos and culture media

Normalized gene expression values (Δ*Ct*) were analyzed using a general linear model including the fixed effects of the pool and the type of embryo (blastocyst or degenerate; in addition to control for media samples). Association between the normalized gene expression and the type of embryo was tested using a likelihood ratio test by comparing to a reduced model without the embryo effect. Threshold for detection was set as *C*t < 34. The mean and the range of the fold change for each miRNA was calculated as 2^−ΔΔ*Ct*^ using the estimated ΔΔ*Ct* value ± standard error (Livak and Schmittgen, 2001).

## Results and discussion

In the present study, the expression of five miRNAs was analyzed for the association with embryo quality in bovine pre-implantation embryos and the presence in bovine and human embryo culture media. Differential expression of miRNAs was found to be associated with embryo quality of day 8 bovine pre-implantation embryos. Additionally, this study is the first to identify the presence of miRNAs in *in vitro* embryo culture media from bovine and human embryos and thus may provide grounds for future development of non-invasive prediction of embryo quality based on miRNA biomarkers.

### Differential expression of miRNAs between blastocysts and degenerate embryos

**Figure 2:**
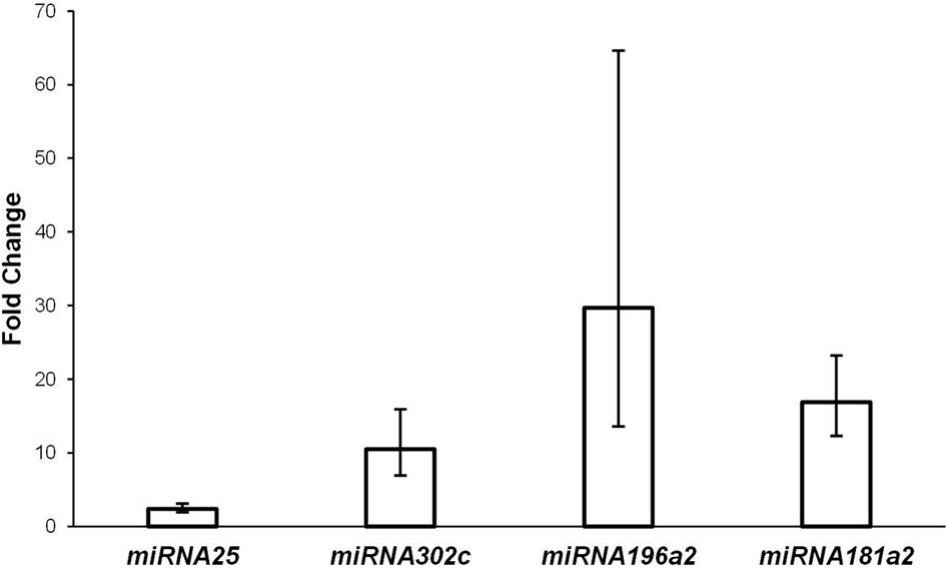
**Relative fold change difference in gene expression of degenerate to blastocyst embryos**.

Gene expression analysis in degenerate vs. blastocyst embryos revealed differential expression in four of five miRNAs tested. MiRNAs miR-181a2 (*P* < 0.0001), miR-196a2 (*P* < 0.0001), miR-302c (*P* < 0.0001), and miR-25 (*P* = 0.002) showed higher expression in degenerate embryos compared to blastocyst embryos (Figure 2). The fold differences in expression between degenerate embryos and blastocysts ranged from 2.4 to 29.7 (Figure 2). In contrast, miR-370 was not differentially expressed between degenerate and blastocyst embryos (*P* < 0.239). Given the observation of significant changes in miRNA expression between embryos of varying quality, it can be inferred that the underlying genetics of miRNA machinery varies greatly between embryos of differing developmental competence.

Functionally, the miRNA tested here have been shown to have roles in embryonic development that could drive developmental outcomes. For instance, miR-196a2 is highly expressed within degenerate embryos compared to blastocyst embryos where it was previously shown that miR-196a2 was overexpressed early in development within bovine embryos followed by a decline from the morula stage (Tripurani et al., 2011). The higher expression observed in degenerates confers dysregulation in these embryos, which may have downstream effects on target genes of the miRNA. Testing of a known bovine target gene, NOBOX, was not found to be detected in these embryos (unpublished data) though other target genes may be affected. Other mRNA targets include Hox genes, which have important roles in limb development and implantation (Chen et al., 2011). HOX genes have complementary binding sites to miR-196a2, where it was shown that miR-196 binds with high complementarity to HOXB8 to cleave the mRNA (Yekta et al., 2004). Given a higher expression of mi196a2 in degenerates, downregulation of genes patterning development could arise in abnormal development of the embryo.

Another previously identified miRNA in bovine embryos is miR-181a as reported by Lingenfelter et al. (2011). It was observed that in embryonic development, miR-181a was highly expressed within the bovine embryo at the 4–8 cell stage and then declines. In our study, the observed increased expression of miR-181a in degenerate embryos may suggest that this miRNA is dysregulated leading to improper progression of development. The study by Lingenfelter et al. (2011) also demonstrates that cells expressing miR-181a reduced the expression of nucleoplasmin 2(NPM2) involved in nuclear organization, a crucial process in fertilization and development. A study which supplemented nucleoplasmin to nuclear transfer (NT) bovine embryos found that pregnancy initiation rates increased for NT embryos while the rate of live births did not (Betthauser et al., 2006). Gene expression profiles of the injected NT embryos was more similar to that of an *in vitro* produced bovine embryo, thus suggesting that nucleoplasmins have roles in reprogramming of the cell. In the present study, miR-181a may regulate NPM2, which in turn alters the developmental competence of the embryo.

Other miRNAs analyzed in embryos of varying quality in this study have been shown to have various roles in cellular reprogramming. Lu et al. (2012) reported that miR-25 expression was found to affect the regulation of genes Wwp2 and Fbxw7, involved in regulating reprogramming of a cell to promote iPSCs, as these genes act to degrade the Yamanaka factors Oct4 and Klf5, respectively. Other studies found that overexpression of miR-25 promoted cell proliferation (Zhang et al., 2012), while its downregulation stimulated apoptosis (Zhang et al., 2012) and oxidative stress (Varga et al., 2013).

Similarly, miR-302c has also been shown to modulate cell reprogramming. Lee et al. (2013) identified methyl-DNA binding domain protein 2 (MBD2) as a direct target of miR-302c. When miR-302c is overexpressed, MBD2 is suppressed resulting in increased NANOG expression promoting reprogramming of a partially reprogrammed cell to a complete reprogrammed cell. Interestingly, miR-302c was induced within stem cell reprogramming experiments in hypoxic conditions and supplementation of media with FGF2. Indeed, it is important to note that culture conditions may cause altered expression of miRNAs as *in vitro* production of embryos relies on the media to support the growing embryo. Overall, miRNAs analyzed in this study were chosen based on known functions in embryonic development, however, many target genes might not be annotated or associated to a given function in development. Here, relatively large changes in expression may correspond to a high degree of difference in the biological mechanisms driving embryonic development as one miRNA can regulate a vast number of mRNA targets. As targets of miRNAs in other tissues and cells are elucidated, their role in embryonic development may be inferred. Thus, future studies should focus on characterization of miRNA expression and pathways enriched between embryos of varying developmental competencies.

### miRNA expression in culture media

Table 3 shows that miR-25 was detected in media derived from bovine blastocysts and degenerates whereas miR-302c was not expressed in the culture media of these two embryo pools. Both miR-25 and miR-302c were not detected in control SOF media regardless of supplementation with BSA (Table 3). These miRNAs were additionally tested in a pool (*n* = 5) day 5 human embryo media drops where a similar expression was observed. Moreover, media drops from day 6 single human embryos were tested and the results paralleled that of both bovine and pooled day 5 human media expression (data not shown).

**Table 3:** **miRNA expression in bovine IVF culture media**.

	**SOF media +BSA**	**Control SOF media +BSA**	**SOF media −BSA**	**Control SOF media −BSA**
miR-25	B/D	–	B/D	–
miR-302c	–	–	–	–
miR-196a2	D	+	–	–
miR-181a	B/D	+	N/A	+
miR-370	B/D	+	B/D	+

*B, blastocyst media; D, degenerate media; –, not expressed; +, expressed*.

Although both miR-25 and miR-302c were expressed within bovine embryos, only miR-25 could be detected in the IVF culture media and the expression of these miRNAs was further observed in single and pooled human derived embryo media. Thus, assessment of miRNA expression in both bovine and human embryos suggests that an embryo may selectively secrete certain miRNAs into their extracellular environment. Indeed, it has been reported that the intracellular and extracellular miRNA spectra is typically cell-type-dependent. For example, Lim et al. (2005) reported that upon delivery of miR-124 to HeLa cells the mRNA expression profile was similar to that of the brain where miR-124 is preferentially expressed, thereby showing that tissue- or cell-type specific miRNA expression may maintain a given cell type. In a study by Valadi et al. (2007) it was found that exosomal miRNA in the extracellular environment were expressed from a limited number of genes. Also, Wang et al. (2010) found cells release many miRNAs, though the intracellular and extracellular spectra of miRNAs vary. Collectively, these studies suggest that miRNAs are cell-type specific and that they may be selectively secreted to represent the differences observed in intracellular and extracellular spectra, similar to the observations in the present study.

Surprisingly, expression of miR-196a2 was detected in degenerate and control media in the presence of BSA, though not statistically different, whereas miR-196a2 expression was not detected in blastocyst media (Table 3). In experiment B, BSA was not supplemented and expression was not detected in neither media derived from embryos nor in the control media. These data suggest that miR-196a2 expression is correlated to BSA supplementation, a novel finding that sheds light on the potential importance of embryo culture media additives.

The data of the present study suggest that media components may be potential miRNA carriers. Results revealed presence of miR-196a2 in the control baseline SOF media when BSA was added; however, it was not detected when BSA was removed from the media. Fraction V BSA is added into our IVF culture system as a protein source where the additive is a highly purified fatty acid free form. In blood, serum proteins act as carriers of many small molecules, therefore we propose that miR-196a2 may bind to BSA for protection and stability. Indeed, recent studies suggest that miRNAs in the extracellular environment are predominantly bound to proteins and not carried by cell-derived vesicles (Wang et al., 2010; Turchinovich et al., 2011). Wang et al. (2010) reported that a significant number of RNA-binding proteins including nucleophosmin1 (NPM1) is released into the culture media of human cell lines. Interestingly, they found that NPM1 forms a RNA-protein complex with miRNA and suggested that this protein may play a role in exportation and protection of miRNAs from degradation. Similarly, Turchinovich et al. (2011) isolated miRNA bound to Ago2 protein, a part of the RNA-induced silencing complex, and suggested that the Ago2/miRNA complex is highly stable following cell lysis for a prolonged period of time. Importantly, a recent study by Xia et al. (2010) provides evidence for the binding of BSA to RNA molecules. The authors analyzed single- and double- stranded oligonucleotide (21 nucleotides in length) binding capacity to BSA and found that BSA can distinguish between the oligonucleotides and is capable of binding to single stranded oligonucleotides through non-specific interaction (Xia et al., 2010). Thus, the aforementioned studies support our hypothesis that the BSA protein added to our system is likely an external source carrying miRNAs.

Differential expression of miR-196a2 in the media from control, blastocysts, and degenerate embryos in the presence of BSA may reflect altered underlying cellular responses. In theory, blastocyst cells may potentially take up the miRNA present in the media at the time of transfer into fresh SOF media on day 5 of development. It has been proposed that miRNAs play a role in cellular communication through exosome mediated transfer of miRNAs (Valadi et al., 2007). Though here we propose BSA to be a protein carrier of miRNAs, the mechanism of cell communication by miRNA-protein complexes has not been established. However, steroid hormone-BSA conjugate molecules have been used to identify non-genomic activities and action at the cell membrane. In a study by Nishimura and Nakano (2000), it was shown that testosterone-bound BSA was found to not only enter the cell but also enter into the nuclei of both spermatocytes and spermatids. Thus, it is plausible that BSA-bound miRNAs may enter into embryonic cells therefore explaining the clearance of miR-196a2 from the media. Conversely, the blastocyst may secrete a substance which acts to degrade the miRNA or the BSA in the media through an unknown mechanism.

MiR-181 and miR-370 were found to be expressed in the control media regardless of BSA supplementation (Table 3). In BSA supplemented media, the expression of both miR-181 and miR-370 was detected though not statistically significantly different between embryos of varying quality or above the baseline control. The expression of miR-370 in blastocyst, degenerate, and control media was not statistically different though there was a tendency for higher expression in degenerate media as the fold change was 4.2 (range 1.78–10.04; *P* = 0.110). When BSA was not supplemented in SOF media, miR-181 showed expression though right at threshold cut off in one blastocyst pool and was not detected in the second pool making the data not conclusive. In contrast, miR-370 was expressed in blastocyst and degenerate media when BSA was not supplemented. As both miR-181a and miR-370 reveal expression in the control media it may be inferred that another source of miRNAs is present in the media.

Differential supplementation of BSA allowed for the isolation of a media component likely to be a source of an external miRNA; however, other components could also be a source of miRNA. MiR-181a and miR-370 were expressed in both the control media supplemented with and lacking BSA. As the SOF media components are often proprietary further analysis should be carried out in investigating carriers of miRNA in the base media as well as supplements. Here we tested BSA; however, other components to test in the future could include the use of antibiotics within the IVF culture system. The results for both miR-181a and miR-370 were rather non-conclusive though further testing could potentially isolate another uncharacterized carrier of miRNA.

## Conclusions

In summary, our study is the first to identify the presence of miRNAs in *in vitro* human and bovine culture media. MiRNAs may be selectively secreted from the embryo, though the expression of the miRNA did not correlate to the degree of gene expression observed within the embryo. Embryo quality or developmental competence was found to be correlated with miRNA expression, where future studies should focus on further profiling miRNAs within embryos of varying quality. As these miRNAs are secreted into the extracellular environment, characterization of expression within embryos and association of expression in the media could allow for development of biomarkers for selection of better quality embryos. Moreover, here we identify that external sources of miRNAs may be added into the media to affect the extracellular environment of the embryo. Though the direct effect on the embryo was not studied, we propose that external sources may be selectively taken up by the embryo. A limitation of this study is the small number of miRNAs used in the culture media and embryos. Therefore, future studies should further investigate the relationship between presence of miRNAs in the media and embryo development using genome-wide screening approaches.

### Conflict of interest statement

The authors declare that the research was conducted in the absence of any commercial or financial relationships that could be construed as a potential conflict of interest.
